# Association of Common Genetic Risk Variants With Gestational Diabetes Mellitus and Their Role in GDM Prediction

**DOI:** 10.3389/fendo.2021.628582

**Published:** 2021-04-19

**Authors:** Polina V. Popova, Alexandra A. Klyushina, Lyudmila B. Vasilyeva, Alexandra S. Tkachuk, Elena A. Vasukova, Anna D. Anopova, Evgenii A. Pustozerov, Inga V. Gorelova, Ekaterina N. Kravchuk, O. Li, Tatiana M. Pervunina, Anna A. Kostareva, Elena N. Grineva

**Affiliations:** ^1^ Almazov National Medical Research Centre, Saint Petersburg, Russia; ^2^ Department of Internal Diseases and Endocrinology, St. Petersburg Pavlov State Medical University, Saint Petersburg, Russia; ^3^ Department of Biomedical Engineering, Saint Petersburg State Electrotechnical University, Saint Petersburg, Russia

**Keywords:** genetics, gestational diabetes mellitus, GDM prediction, single nucleotide polymorphism, genetic risk score

## Abstract

**Objective:**

We aimed to explore the associations between common genetic risk variants with gestational diabetes mellitus (GDM) risk in Russian women and to assess their utility in the identification of GDM cases.

**Methods:**

We conducted a case-control study including 1,142 pregnant women (688 GDM cases and 454 controls) enrolled at Almazov National Medical Research Centre. The International Association of Diabetes and Pregnancy Study Groups criteria were used to diagnose GDM. A total of 11 single- nucleotide polymorphisms (SNPs), including those in *HKDC1* (rs10762264), *GCK* (rs1799884), *MTNR1B* (rs10830963 and rs1387153), *TCF7L2* (rs7903146 and rs12255372), *KCNJ11* (rs5219), *IGF2BP2* (rs4402960), *IRS1* (rs1801278), *FTO* (rs9939609), and *CDKAL1* (rs7754840) were genotyped using Taqman assays. A logistic regression model was used to calculate odds ratios (ORs) and their confidence intervals (CIs). A simple-count genetic risk score (GRS) was calculated using 6 SNPs. The area under the receiver operating characteristic curve (c-statistic) was calculated for the logistic regression model predicting the risk of GDM using clinical covariates, SNPs that had shown a significant association with GDM in our study, GRS, and their combinations.

**Results:**

Two variants in *MTNR1B* (rs1387153 and rs10830963) demonstrated a significant association with an increased risk of GDM. The association remained significant after adjustment for age, pre-gestational BMI, arterial hypertension, GDM in history, impaired glucose tolerance, polycystic ovary syndrome, family history of diabetes, and parity (P = 0.001 and P < 0.001, respectively). After being conditioned by each other, the effect of rs1387153 on GDM predisposition weakened while the effect of rs10830963 remained significant (P = 0.004). The risk of GDM was predicted by clinical variables (c-statistic 0.712, 95 % CI: 0.675 – 0.749), and the accuracy of prediction was modestly improved by adding GRS to the model (0.719, 95 % CI 0.682 – 0.755), and more by adding only rs10830963 (0.729, 95 % CI 0.693 – 0.764).

**Conclusion:**

Among 11 SNPs associated with T2D and/or GDM in other populations, we confirmed significant association with GDM for two variants in *MTNR1B* in Russian women. However, these variants showed limited value in the identification of GDM cases.

## Introduction

Gestational diabetes mellitus (GDM) is a highly prevalent condition affecting 9.3-25.5% of pregnant women (1). GDM is associated with considerable adverse pregnancy outcomes, including birth trauma (2), increased caesarean delivery rate, as well as the future development of type 2 diabetes (T2D) both in the mother and in the offspring (3, 4).

The pathogenesis of GDM is similar to T2DM, as both conditions are characterized by insulin resistance and a compensatory increase in insulin secretion that is unable to meet requirements (5).

A shared genetic background for GDM and T2DM has been proposed due to the common family history (6) and due to the association of GDM with the increased likelihood of developing T2DM later in life (7).

There are over 160 genetic loci that have been associated with T2DM in non-pregnant population (8) and a limited number of them have been evaluated in pregnant women (9). By the time of planning of this study there were two published meta-analyses confirming association of the following variants with GDM: melatonin receptor 1B *(MTNR1B)*, glucokinase (*GCK)*, transcription factor 7-like 2 (*TCF7L2)*, potassium inwardly rectifying channel, subfamily J, member 11 (*KCNJ11)*, regulatory subunit associated protein 1-like 1 *(CDKAL1)*, insulin-like growth factor 2 mRNA-binding protein 2 *(IGF2BP2)* and insulin receptor substrate 1 (*IRS1*) (10, 11).

However, the association between specific gene with the risk of a certain disease may considerably vary among different ethnicities. Indeed, in replication studies, similar effects in different ethnic populations were confirmed only for a part of these risk alleles (12–17). To the best of our knowledge, there have been no studies on the association between common genetic risk variants with GDM risk in Russian population except for our previous study which was limited by a relatively small sample size (18). Therefore, we aimed to study the associations between the above mentioned SNPs with GDM risk in Russian population with an extended sample size.

We have also added to the panel rs10762264 in *hexokinase domain containing 1 (HKDC1)* and rs9939609 in fat mass and obesity-associated protein (*FTO)*. Variants in *HKDC1*, which is related to pathways of carbon metabolism, have been identified to be connected to 2-h plasma glucose (2HPG) in pregnancy in a genome-wide association study of 4,437 pregnant mothers of European, Thai, Afro-Caribbean, and Hispanic ancestry (19).

The secondary aim of our study was to explore the hypothesis that the addition of the significant genetic variants will increase the accuracy of the model identifying GDM cases compared to the model based on solely clinical parameters.

## Methods

This case-control study included participants of prospective hospital-based cohort of pregnant women screened for GDM at the Almazov National Medical Research Centre (NMRC) from January 2012 to December 2014 and participants of GEM-GDM study performed at the Almazov NMRC from July 2015 to July 2020 (20). A total of 688 women with GDM and 454 controls were randomly selected from the two cohorts. The majority of the participants were ethnic Russians. We included women with singleton pregnancy aged 18-45 years. Women with pre-gestational diabetes, diseases affecting carbohydrate metabolism, and fasting glucose levels >7.0 mmol/L were excluded.

The ethical committee of the Almazov NMRC reviewed and approved the study protocol (protocol no. 119). The written informed consent was signed by all the participants. The study was conducted in accordance with the Declaration of Helsinki.

The 2-hour oral glucose tolerance test (OGTT) with 75-g glucose was performed in the 24^th^-28^th^ week of gestation. Plasma glucose (PG) concentration was determined by the glucose oxidase method in fresh plasma samples.

The International Association of Diabetes and Pregnancy Study Groups (IADPSG) criteria were used for the diagnosis of GDM (fasting glucose of ≥5.1 mmol/L, and/or postprandial glucose of ≥10.0 mmol/L after 1 h, and/or ≥8.5 mmol/L after 2 h) (21). Pregnant women with normal glucose tolerance were included as controls.

Blood for genotyping of pregnant women and serum for biochemical analysis were obtained during OGTT and stored at -80°C until the analysis. Serum fasting insulin levels were measured using the electrochemiluminescence immunoassay (Roche Diagnostics, GmbH, Germany).

Homeostatic model assessment (HOMA) index was calculated using the following formula: fasting serum insulin (m IU/L) × fasting plasma glucose (mmol/L)/(22.5) as an insulin resistance indicator.

The following data were collected from medical charts: arterial hypertension, GDM in history, impaired glucose tolerance, polycystic ovary syndrome, family history of diabetes, parity, pre-gestational weight and blood pressure measured at the time of OGTT. Pre-gestational body mass index (BMI) was calculated by dividing weight (in kilograms) by the square of height (in meters).

### DNA and Genotyping

Genomic DNA was isolated from blood using the FlexiGene DNA Kit (Qiagen, Hilden, Germany). The variants of *HKDC1 (*rs10762264), *MTNR1B* (rs10830963 and rs1387153), *GCK* (rs1799884), *KCNJ11* (rs5219), *IGF2BP2* (rs4402960), *TCF7L2* (rs7903146), *CDKAL1* (rs7754840), *FTO* (rs9939609) and *IRS1* (rs1801278) were genotyped by real-time PCR with custom kits (Applied Biosystems, USA), following procedures recommended by the manufacturer. Each primer tube contains a concentrated mixture of SNP Genotyping Assay Mix, which includes polymorphism-specific direct and reverse primers, two TaqMan MGB probes: tagged with VIC dye to identify allele 1, and tagged with FAM dye to identify allele 2. After the replication of 10% of the samples the discordance rate was found to be less than 0.1%.

### Statistical Analyses

The sample size was calculated using G*Power 3.1. A total of 1135 participants are required to have a 85% chance of detecting, as significant at the 0.008 level, an increase in the primary outcome measure from 9.5% in the control group to 17.6% in the GDM group (based on our previous results) (18). The level of significance of 0.008 was selected because we planned to check association of GDM with 6 SNPs with Bonfferroni correction (0.05/6).

Statistical analyses were performed in SPSS (Chicago, IL, USA) version 22.0. The data are presented as the mean ± standard deviation. The χ^2^ criterion was used to compare the distribution of qualitative characteristics. Differences in the quantitative characteristics of the groups were assessed with Student’s t-test. A two-sided P-value <0.05 was considered statistically significant.

To estimate the individual effect of each variant (using 0, 1 and 2 allelic count) on the risk of gestational diabetes, odds ratios (OR) and 95% confidence interval (95% CI) were calculated with logistic regression analysis (Binary logistic regression, forward conditional). Maternal age, pre-gestational BMI, arterial hypertension, GDM in history, impaired glucose tolerance, polycystic ovary syndrome, family history of diabetes, and parity were included as covariates in the logistic regression model. Additional logistic regression model was built including the listed above parameters and HOMA index measured at the time of OGTT.

We also assessed the cumulative effect on GDM risk of the combination of SNPs genotyped in the majority of the participants (>90%). For this purpose, we calculated genetic risk score (GRS) as a sum of each risk alleles (0, 1 or 2) for the following variants: rs10830963 in *MTNR1B* (risk allele *G) (29)*, rs7754840 in *CDKAL1 (C)*, rs1799884 in *GCK (T) (24)*, rs5219 in *KCNJ11 (T) (29)*, rs4402960 in *IGF2BP2(T) (29)*, and rs7903146 in *TCF7L2 (T) (24).*

When calculating the GRS in patients with incomplete genotypes we assumed that they did not carry the risk allele (i.e., we assigned a score of 0 for the missing genotype). While performing a sensitivity analysis, patients with missing genotypes were excluded.

The area under the receiver-operating characteristic curve (c-statistic) was calculated for the logistic regression model predicting the risk of GDM using clinical covariates, rs10830963, GRS or their combinations.

## Results

A total of 1142 pregnant women including 688 patients with GDM and 454 individuals with normal glucose tolerance (controls) were included in this study. The clinical and biochemical characteristics of the two groups are presented in **Table 1**. Women with GDM were older and had a higher pre-pregnancy BMI (p<0.0001 for both comparisons). There was a higher frequency of impaired glucose tolerance (IGT), arterial hypertension, GDM in history and family history of type 2 diabetes mellitus in GDM group compared to control group. Women with GDM had higher levels of systolic and diastolic blood pressure (BP), as well as higher levels of fasting insulin, HOMA index, higher levels of fasting, 1-h and 2-h plasma glucose in OGTT. There was a higher percentage of multiparae in the GDM group.

**Table 1:** Clinical and biochemical characteristics of GDM patients and controls.

	GDM N=688	Control N=454	Р
Age, years	31.9 ± 4.5	29.5 ± 4.7	<0.0001
Pre-pregnancy BMI, kg/m^2^	25.1 ± 5.6	23.0 ± 4.6	<0.0001
Family history of diabetes (%)	44.4 %	37.5 %	0.023
History of arterial hypertension (%)	8.8 %	7.4 %	0.439
History of GDM (%)	11.9 %	0.8 %	<0.001
History of IGT (%)	4.2 %	1.8 %	0.026
PCOS (%)	8.5 %	6.3 %	0.207
Parity:			0.001
Nulliparae (%)	32.8 %	43.9 %	
Multiparae (%)	67.2 %	56.1 %	
Number of pregnancies*	2.3 ± 1.7	1.5 ± 1.5	<0.001
Systolic BP (mm Hg)	118.7 ± 12.2	112.8 ± 11.0	<0.001
Diastolic BP (mm Hg)	74.6 ± 9.0	71.7 ± 9.2	<0.001
Fasting plasma glucose (mmol/L)	5.1 ± 0.6	4.3 ± 0.5	<0.0001
1-h postload glucose (mmol/L)	9.4 ± 1.8	6.9 ± 1.4	<0.0001
2-h postload glucose (mmol/L)	8.3 ± 1.8	5.9 ± 1.2	<0.0001
Insulin, m IU/L**	14.2 ± 10.6	10.6 ± 6.1	<0.0001
HOMA**	3.3 ± 2.9	2.2 ± 1.3	<0.0001

BMI, body mass index; GDM, gestational diabetes mellitus; IGT, impaired glucose tolerance; PCOS, polycystic ovary syndrome; BP, blood pressure.

*including the index pregnancy.

**analysis performed in 374 patients with GDM and 126 women from control group.

The results of genotyping are shown in **Table 2**. We observed significant differences in the distribution of the rs10830963 and rs1387153 in MTNR1B gene between GDM patients and controls. The genotype distributions of the studied single-nucleotide polymorphisms (SNPs) were all in Hardy-Weinberg equilibrium (P > 0.05). The TT genotype of IRS1 rs1801278 was not detected in this population.

**Table 2:** Genotype and allele distribution among GDM patients and controls.

Gene	Variants, minor allele	N (GDM/control)	Genotypes in controls N (%)			Genotypes in GDM patients N (%)			Р*
			AA	AB	BB	AA	AB	BB	
*MTNR1B*	rs10830963, G	688/454	36 (7.9)	185 (40.7)	233 (51.3)	109 (15.8)	351 (51.0)	228 (33.1)	<0.001
	rs1387153, T	320/318	19 (6.0)	134 (42.1)	165 (51.9)	51 (15.9)	147 (45.5)	122 (38.1)	<0.001
*HKDC1*	rs10762264, G	432/157	16 (10.2)	68 (43.3)	73 (46.5)	44 (10.2)	178 (41.2)	210 (48.6)	0.892
*CDKAL1*	rs7754840, C	685/450	43 (9.6)	207 (46.0)	200 (44.4)	84 (12.3)	286 (41.8)	315 (46.0)	0.218
*GCK*	rs1799884, T	688/454	12 (2.6)	99 (21.8)	343 (75.6)	27 (3.9)	173 (25.1)	488 (70.9)	0.183
*IRS1*	rs1801278, T	319/318	0 (0)	34 (10.7)	284 (89.3)	0 (0)	27 (8.5)	292 (91.5)	0.350
*KCNJ11*	rs5219, T	684/450	74 (16.4)	217 (48.2)	159 (35.3)	132 (19.3)	292 (42.7)	260 (38.0)	0.166
*IGF2BP2*	rs4402960, T	686/450	53 (11.8)	193 (42.9)	204 (45.3)	61 (8.9)	322 (46.9)	303 (44.2)	0.190
*TCF7L2*	rs7903146, T	684/449	27 (6.0)	154 (34.3)	268 (59.7)	42 (6.1)	255 (37.3)	387 (56.6)	0.569
	rs12255372, T	295/191	11 (5.8)	61 (31.9)	119 (62.3)	21 (7.1)	93 (31.5)	181 (61.4)	0.840
*FTO*	rs9939609, A	290/190	31 (16.3)	94 (49.5)	65 (34.2)	65 (22.4)	140 (48.3)	85 (29.3)	0.218

*P-value of two-sided chi-squared test for comparison of genotypes between the GDM and control groups.

The association of the T allele of rs1387153 and the G allele of rs10830963 with the high GDM risk was confirmed by the logistic regression analysis. Moreover, this association remained significant after adjustment for pre-pregnancy BMI, age, arterial hypertension, GDM in history, IGT, polycystic ovary syndrome, family history of diabetes, parity and HOMA index (**Table 3**).

**Table 3:** Association of SNPs in *MTNR1B* with GDM risk.

Variant	Genotype	OR (95% CI)	P	OR (95% CI)*	P*	OR (95% CI)**	P**
rs10830963	CC	1		1		1	
	GC	1.9 (1.5 – 2.5)	<0.001	2.2 (1.5 – 3.0)	<0.001	1.9 (1.2 – 3.1)	0.005
	GG	3.1 (2.0 – 4.7)	<0.001	2.7 (1.6 – 4.7)	<0.001	3.3 (1.5 – 7.7)	0.005
rs1387153	CC	1		1		1	
	CT	1.5 (1.1 – 2.1)	0.019	1.8 (1.1 – 2.8)	0.022	2.1 (1.1 – 3.9)	0.018
	TT	3.6 (2.0 – 6.4)	<0.001	4.2 (1.8 – 9.7)	0.001	5.8 (1.6-21.0)	0.007

*Logistic regression analyses adjusted for age, pre-gestational BMI, arterial hypertension, GDM in history, impaired glucose tolerance, polycystic ovary syndrome, family history of diabetes, and parity.

**Logistic regression analyses adjusted for the listed above variables and HOMA index.

In order to assess the independent influence of each SNP on GDM risk, conditional logistic regression analysis was performed. After being conditioned by each other, the effect of rs1387153 on GDM predisposition weakened while the effect of rs10830963 remained significant (P = 0.004) and increased with the increase of the number of minor alleles G (OR = 2.6, 95 % CI: 1.5-4.6 for GC genotype and OR = 3.0, 95 % CI: 1.3-7.1 for GG vs CC genotype).

**Figure 1:**
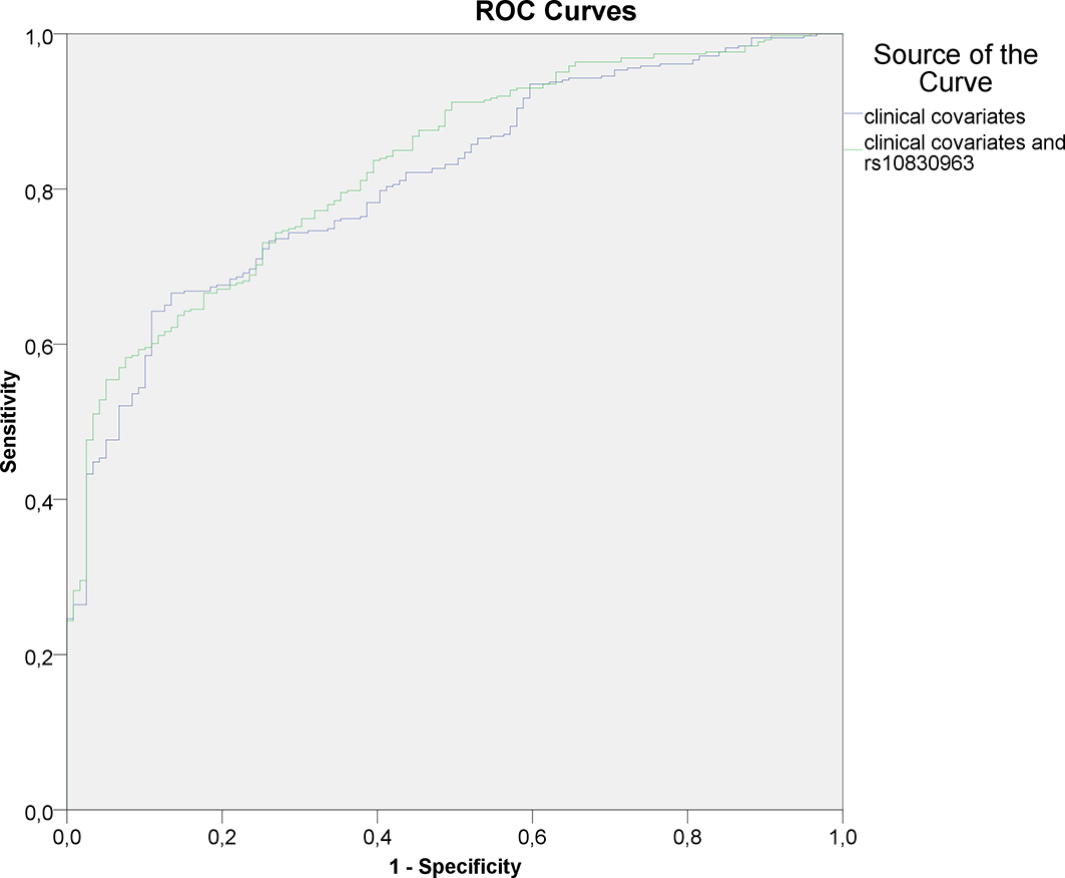
ROC-curves for logistic regression models utilizing clinical covariates (including HOMA index) and rs10830963 as input variables.

The c-statistic for logistic regression models were as follows; clinical covariates only: 0.712 (95 % CI: 0.675 – 0.749), clinical covariates and HOMA index - 0,812 (95% CI 0,772-0,851), GRS only: 0.563 (95 % CI 0.529 – 0.597), rs10830963 only: 0.603 (95 % CI: 0.570 – 0.636), combination of clinical covariates and GRS: 0.719 (95 % CI 0.682 – 0.755); combination of clinical covariates, HOMA index and GRS: 0.822 (95% CI 0.783-0.861); combination of clinical covariates and rs10830963: 0.729 (95 % CI 0.693 – 0.764); combination of clinical covariates, HOMA index and rs10830963: 0.830 (95% CI 0.792 - 0.868). The appropriate ROC-curves for models utilizing clinical data plus HOMA index and clinical data plus HOMA index combined with rs10830963 are shown in **Figure 1**.

## Discussion

Our case-control study confirmed the association of two SNPs in MTNR1B (rs10830963 and rs1387153) with the risk of GDM in Russian women. However, the incorporation of these SNPs into the model predicting GDM did not substantially increase the accuracy. Surprisingly, we didn’t confirm our previous finding of association between rs1799884 in GCK and GDM risk (18).

Our observations are in line with the other studies which have shown significant association of SNPs rs10830963 and rs1387153 in MTNR1B with GDM risk (22–24). The SNP rs10830963 was one of the two variants associated with GDM in GWAS performed in Korean women (22). Furthermore, a large meta-analysis addressing the association of six T2D risk variants with GDM demonstrated that rs10830963 was most strongly associated with GDM risk (23). Recently, a large study including 2,636 GDM cases and 6,086 controls from the Nurses’ Health Study II (NHSII) and the Danish National Birth Cohort (DNBC) confirmed the association of rs10830963 with GDM risk (24).

MTNR1B is a receptor of melatonin, which is involved in the regulation of circadian rhythms and their interaction with physiological functions (including glucose homeostasis) (25). MTNR1B is expressed in various cells and tissues, including pancreatic beta cells (26). The two genetic variants of MTNR1B described in this study are involved in glucose metabolism by reducing early insulin secretion through several parallel signaling pathways in pancreatic beta cells (26, 27).

Our findings that the addition of genetic information to clinical variables improved the prediction of GDM modestly are consistent with several other studies (28, 29). In a recent study by Kawai L. et al. incorporation of the genetic risk score (GRS) composed of 34 T2DM associated variants increased the predictive ability of the clinical model from 0.67 to 0.70 (c- statistic) (29) It is probably due to the fact that clinical risk factors themselves have a genetic component that is in part depicted by the GRS.

Our findings show that genetic risk score does not substantially add power to GDM prediction if compared to clinical characteristics of women, that remain essential for GDM screening and diagnosis.

Among clinical factors associated with GDM in previous studies (30–33) we confirmed the significance of age, systolic BP, pre-gestational BMI, the presence of arterial hypertension in history, GDM in history, parity and HOMA index. However, we did not observe association of PCOS, IGT in history and T2DM in family history with GDM risk in adjusted model. This may be due to a low frequency of PCOS and IGT in the study population with a substantial part of cases remaining undiagnosed.

We did not confirm associations with GDM of loci connected to GDM elsewhere, including variants in the *HKDC1 (*rs10762264), *GCK* (rs1799884), *KCNJ11* (rs5219), *IGF2BP2* (rs4402960), *TCF7L2* (rs7903146), *CDKAL1* (rs7754840), *FTO* (rs9939609) and *IRS1* (rs1801278).

These negative findings could result from population-based differences or could be due to limited statistical power of our study to detect association of GDM with SNPs which have small effects. The previously reported T2D loci had low effect sizes, usually under odds ratios of 1.2, which our study was not powered to detect.

Alternately, as there is no worldwide consensus for GDM diagnostic criteria, it could be due to different thresholds since some women could be classified as controls based on different criteria.

However, our data are supported by a recent meta-analysis which comprehensively quantified the association between the *IGF2BP2* rs4402960 polymorphism and GDM risk and with sufficient statistical evidence supported the null association (34).

Our study has several strengths and limitations which should be considered when interpreting the results. One of the strengths of the study is the well-documented diagnosis of GDM acquired from medical records, which minimized potential disease misclassification. In addition, our participants were well characterized clinically, enabling us to examine whether the SNPs-GDM associations were modified by other major risk factors of GDM. Several clinical parameters, including age, BMI, parity and HOMA index were not equally distributed between cases and controls; however, it was adjusted in each analyzed model.

Although we adjusted for parity, we cannot exclude the possibility that some control women may develop GDM in future pregnancies and could be misclassified in the present study. Another limitation is that we included only candidate SNPs previously known to be associated with GDM without discovering novel variants of GDM risk. Finally, we cannot exclude the weak effects of SNPs on GDM which may not have been detected.

Taking into consideration the high cost of genetic testing and the limited value of GRS in the identification of GDM cases, we would not recommend routine use of GRS for the prediction of GDM. However, GWAS or whole genome sequencing could facilitate unraveling the genetic basis of GDM in Russia.

## Data Availability Statement

The raw data supporting the conclusions of this article will be made available by the authors, without undue reservation.

## Ethics Statement

The studies involving human participants were reviewed and approved by The ethics committee of the Almazov National Medical Research Centre. The patients/participants provided their written informed consent to participate in this study.

## Author Contributions

Conceptualization: PP, AKо and EG. Methodology: PP and AKo. Investigation: PP, AT, EV, IG, AA and OL. Specimen processing and genotyping assays: AKl, LV, EK. Resources: TP. Data curation: AT, AA and EV. Writing—original draft preparation: PP and EP. Writing—review and editing: PP, EG, AKo and TP. Statistical analysis: PP and EP. Supervision: EG and PP. Project administration: EG. Funding acquisition: PP, EP and EG. All authors contributed to the article and approved the submitted version.

## Funding

This work was financially supported by the Ministry of Science and Higher Education of the Russian Federation (Agreement No. 075-15-2020-901). 

## Conflict of Interest

The authors declare that the research was conducted in the absence of any commercial or financial relationships that could be construed as a potential conflict of interest.
